# Canine eumycetoma caused by *Madurella pseudomycetomatis*

**DOI:** 10.1016/j.mmcr.2022.01.007

**Published:** 2022-02-01

**Authors:** Francesco Albanese, Luisa Vera Muscatello, Alice Michelutti, Christian Falcaro, Laura Bellentani, Patrizia Danesi

**Affiliations:** a MYLAV Veterinary Laboratory, Passirana di Rho, Milan, 20017, Italy; b Department of Veterinary Medical Sciences, University of Bologna, Ozzano dell’Emilia, Bologna, 40064, Italy; c Istituto Zooprofilattico Sperimentale delle Venezie, Legnaro, Padua, 35020, Italy; d Clinica Veterinaria Orobica, Azzano San Paolo, Bergamo, 24052, Italy

**Keywords:** True mycetoma, Subcutaneous, Black-grains, Madurella pseudomycetomatis, Dog

## Abstract

Canine eumycetoma is a rare granulomatous disease caused by dematiaceous fungi.

A 2-year-old Great Dane dog had a subcutaneous mass in the right thigh that was surgically removed. Grossly, numerous black-grains were visible. Histologically subcutaneous pyogranulomas were centered on myriads of pigmented fungal elements. *Madurella pseudomycetomatis* was molecularly characterized.

## 1 Introduction

Mycetoma is a chronic granulomatous inflammatory disease, clinically characterized by tumefaction, multiple draining sinuses, and the presence of grains caused by fungi (eumycetoma) or filamentous bacteria (actinomycetoma). In humans, fungal eumycetoma is classified among the neglected diseases, which, by definition, are conditions that occur in tropical areas and affect people living in poverty. Fungal eumycetoma is extremely rare in animals and has a slow course, characterized by the development of dermal/subcutaneous nodules containing black-grains, packed fungal mycelia embedded in a hard and brown-black cement material [[1], [2], [3], [4], [5]]. In humans, eumycetoma is caused worldwide by *Madurella* species in endemic tropical and subtropical areas (such as India, Africa, and South America), while rarely encountered in Europe. *Curvularia* spp., *Cladophialophora bantiana, Pseudollechiaria bodydii* and *Madurella mycetomatis* are the most common fungi that cause canine eumycetoma, with either subcutaneous, osseous, intrabdominal or disseminated lesions [[1], [2], [3], [4], [5]]. In both hosts the infection is acquired by traumatic skin inoculation of infective spores from the environment. We report the first case of subcutaneous eumycetoma caused by *Madurella pseudomycetomatis* in a dog living in Italy.

## 2 Case presentation

**Fig. 1:**
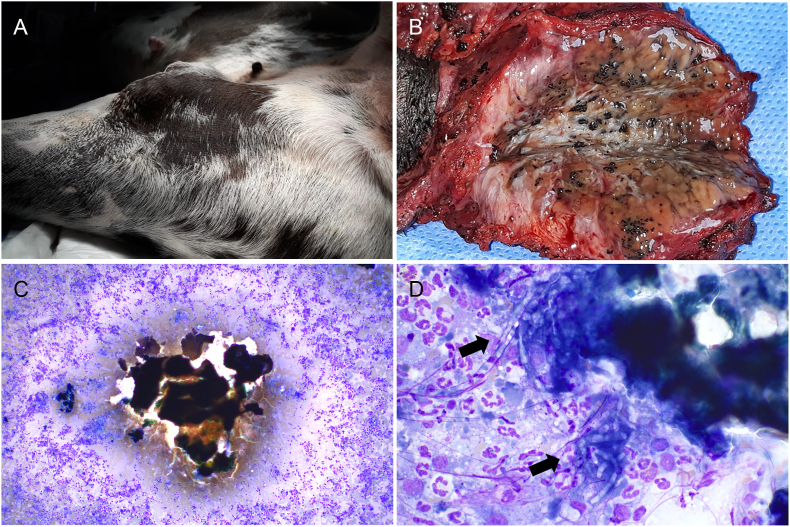
Clinical presentation, macroscopic view of grains, and cytology of black-grain eumycetoma in a Great Dane dog. a) Large, with poorly defined margins, subcutaneous mass in the inner right thigh; b) Sectioned mass, with numerous disseminated black-grains; c) cytology: pyogranulomatous inflammation surrounding a large dark amorphous material (black-grain), 4x; d) Lightly pigmented hyphae are clearly recognizable at the periphery of the grain (arrow), embedded in a neutrophilic and macrophagic inflammation.

A 2-year-old, male, 70 kg, Great Dane dog, underwent on day 0 a clinical examination for a 10 cm subcutaneous mass, with poorly defined margins, in the inner surface of the right thigh (Fig. 1a).

The mass was excised on day 10, and in cut section showed whitish surface with numerous irregular black dot-shaped grains of 0.2–0.6 cm in size (Fig. 1b).

**Fig. 2:**
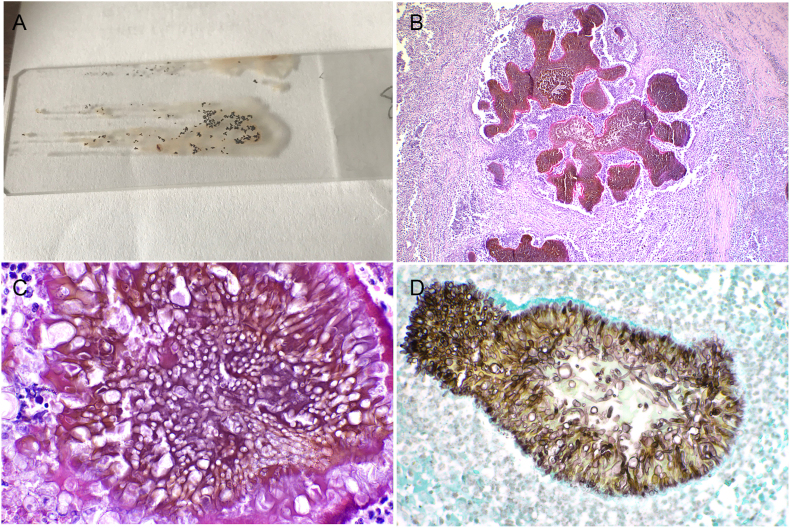
Histological and histochemical features of black-grain eumycetoma in a Great Dane dog. a) Unstained cytological smear showing grossly visible black-grains. b) Pyogranulomas centered on pigmented aggregations of packed fungal hyphae (grains), HE, 4x. c) Pigmented fungi of 7–10 μm in width, infrequently septate, with irregular branching and distorted hyphae, surrounded at the periphery by radially arranged eosinophilic material (Splendor-Hoeppli phenomenon). d) High magnification showing numerous hyphae and roundish large thick-walled chlamydospores-like elements, grocott silver staining, 40x.

Fine needle aspiration cytology revealed many black foci of amorphous material, surrounded by inflammatory cells (Fig. 1c) mostly represented by neutrophils, and from vacuolated to epithelioid macrophages along a few of multinucleated giant cells and lymphocytes. A few septate and branched fungal hyphae, both free and phagocytized by histiocytic cells, were also observed (Fig. 1d). Multiple grains, black in color, were grossly evident on the unstained cytological smear (Fig. 2a).

The mass was submitted for histological examination. The material was formalin-fixed, paraffin embedded (FFPE), and stained with hematoxylin-eosin (HE), grocott silver, and periodic acid shift (PAS) stains.

Histologically, the subcutis was effaced by multifocal nodular pyogranulomas composed of numerous viable and degenerated neutrophils, foamy and epithelioid macrophages, and multinucleated giant. The cells were centered around 0.5–1 mm aggregates (grains; Fig. 2b) of pigmented fungi of 7–10 μm in width, represented by both infrequently septate and with irregular branching and distorted hyphae, and roundish large thick-walled chlamydospores-like elements (Fig. 2c). The fungal aggregates were surrounded at the periphery by radially arranged eosinophilic material (Splendor-Hoeppli phenomenon; Fig. 2c). PAS and Grocott stains further highlighted the fungal elements (Fig. 2d).

A histological diagnosis of severe locally extensive pyogranulomatous panniculitis with intralesional pigmented fungi, suggestive of black-grain eumycetoma, was reached.

Ten micron-thick sections (n=4) were submitted for molecular characterization by using SYBR Green Real-Time PCR (rtPCR) as reported previously [6].

Positive 28S LSU rRNA amplifications were obtained and sequenced from FFPE tissue. *Madurella pseudomycetomatis* was identified. Molecular phylogeny was performed on the 28S LSU rRNA sequence dataset. The rooted tree was constructed including *Madurella pseudomycetomatis* from this study and other Sordariales species (*Madurella*, *Chaetomidium*, *Sordaria*, *Podospora*, *Lasiosphaeria*, and *Schizothecium* species) available from the GeneBank database. Sequences of *Pleospora herbarum* were used as outgroup.

**Fig. 3:**
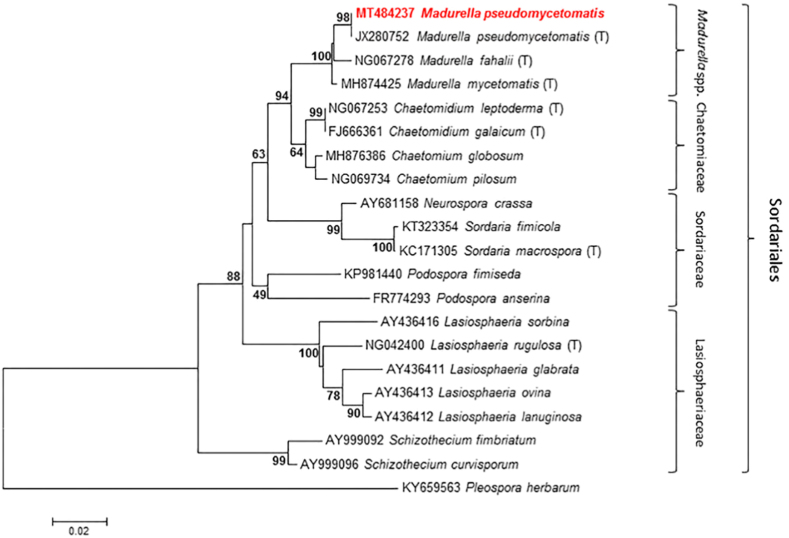
Phylogenetic tree based on 28S LSU sequences of various members of the order Sordariales. Sequences produced from our study are shown in red boldface. *Pleospora herbarum* sequence was used as an outgroup. The tree was constructed using the neighbor joining method. Bootstrap values shown at the main nodes represent the probabilities based on 1000 replicates. (T) = type strains. (For interpretation of the references to color in this figure legend, the reader is referred to the Web version of this article.)

*Madurella pseudomycetomatis* (MT484237) from this study showed 100% similarity with *M. pseudomycetomatis* type strain (JX280752). Phylogenetically, both sequences were monophyletic with related species in the *Madurella* clade (Fig. 3).

Due to the impossibility of surgically removing the entire lesion, a systemic antifungal therapy based on itraconazole (10 mg/Kg/d for 4 weeks) was prescribed on day 10. Considering the clinical improvement and the high cost of the drug, the therapy was continued every other week (pulse therapy) for 45 days until no clinical lesion was grossly appreciable (on day 65).

After two years and seven months (until day 995) no relapse of lesion has occurred.

## 3 Discussion

Black-grain mycetoma is an extremely rare fungal disease and approximately 23 species of fungi have been reported in human literature [7]. The predominant agents worldwide of black-grain mycetoma are affiliated with the genus *Madurella*, in the Sordariales order of Ascomycota (Fig. 3) [7]. This case describes a *M. pseudomycetomatis* canine eumycetoma in Italy (temperate climate region). This disease is unusual because, although rare reports in Europe are occasionally described, it more commonly affects humans and dogs in endemic tropical and subtropical regions [2]. Because in non-endemic areas *Madurella* eumycetoma is rarely described as an autochthonous case, this report greatly improves the epidemiology of this fungi, demonstrating its presence in Italy and the important role of animals as sentinels for human exposure to saprophyte fungi. Regarding this dog, no history of traumatic wounds was described by owner, but the dog was living outdoors, and traumatic inoculation of soil contaminated with mold is the most likely route of infection.

In addition, the molecular characterization performed in this study allowed identification of the new species *Madurella pseudomycetomatis* so far only reported in human literature.

With this report, the presence of *Madurella pseudomycetomatis* should also be considered as possible etiology of canine eumycetoma in dogs.

## Declaration of competing interest

There are none.
